# Optimization of Photosynthetic Photon Flux Density and Light Quality for Increasing Radiation-Use Efficiency in Dwarf Tomato under LED Light at the Vegetative Growth Stage

**DOI:** 10.3390/plants11010121

**Published:** 2021-12-31

**Authors:** Xinglin Ke, Hideo Yoshida, Shoko Hikosaka, Eiji Goto

**Affiliations:** 1 Graduate School of Horticulture, Chiba University, Matsudo 648, Chiba 271-8510, Japan; kexinglin92@outlook.com (X.K.); yoshida.hideo@chiba-u.jp (H.Y.); s-hikosaka@faculty.chiba-u.jp (S.H.); 2 Plant Molecular Research Center, Chiba University, Chiba 260-0856, Japan

**Keywords:** tomato, vertical farming, red light, blue light, white light, leaf optical properties, light interception, photosynthetic light-use efficiency, projected leaf area, chlorophyll concentration

## Abstract

Dwarf tomatoes are advantageous when cultivated in a plant factory with artificial light because they can grow well in a small volume. However, few studies have been reported on cultivation in a controlled environment for improving productivity. We performed two experiments to investigate the effects of photosynthetic photon flux density (PPFD; 300, 500, and 700 μmol m^−2^ s^−1^) with white light and light quality (white, R3B1 (red:blue = 3:1), and R9B1) with a PPFD of 300 μmol m^−2^ s^−1^ on plant growth and radiation-use efficiency (RUE) of a dwarf tomato cultivar (‘Micro-Tom’) at the vegetative growth stage. The results clearly demonstrated that higher PPFD leads to higher dry mass and lower specific leaf area, but it does not affect the stem length. Furthermore, high PPFD increased the photosynthetic rate (Pn) of individual leaves but decreased RUE. A higher blue light proportion inhibited dry mass production with the same intercepted light because the leaves under high blue light proportion had low Pn and photosynthetic light-use efficiency. In conclusion, 300 μmol m^−2^ s^−1^ PPFD and R9B1 are the recommended proper PPFD and light quality, respectively, for ‘Micro-Tom’ cultivation at the vegetative growth stage to increase the RUE.

## 1. Introduction

Plant factories with artificial light (PFALs), also called vertical farms, are becoming popular in many countries for the commercial production of vegetables. Generally, the distance between the adjacent vertical cultivation layers in a PFAL with high space utilization efficiency is approximately 40–50 cm. Therefore, 30 cm or less is the suitable plant height [1]. Dwarf tomatoes have several advantages compared with general tomatoes in terms of their commercial production in a PFAL: small size with adult ‘Micro-Tom’ (the most famous dwarf tomato cultivar) plant height of 15–20 cm; can grow at high plant densities [2]; probably have low light requirements as the net photosynthetic rate (Pn) of Cross no. 2 plants (transgenic tomato bred by cross-breeding line 56B and ‘Micro-Tom’) was saturated at a photosynthetic photon flux density (PPFD) of 400 μmol m^−2^ s^−1^ and CO_2_ concentration of 500 μmol mol^−1^ [3]; they have a short life cycle, which allows harvesting within 70–90 days after sowing [4], which in turn leads to lower production costs and expedites the tomato production process compared with a general tomato cultivar. Owing to these advantages, dwarf tomatoes can be successfully cultivated in PFALs.

In a PFAL, over 50% of the electricity is consumed in lighting [5,6]. Therefore, a more efficient use of light is one approach to reduce electricity costs. Goto [7] proposed that the production efficiency of plants cultivated in a PFAL can be measured as photoassimilates per unit molar number of photons (g mol^−1^). The author calculated the values for rice, strawberry, lettuce, and tomato from the experimental data and indicated that the values did not differ significantly among food crops.

Radiation-use efficiency (RUE) is a good index for measuring radiation utilization by plants [8,9] and an important quantifier of crop production in relation to photosynthesis, as it combines both the amount of radiation captured by the crop and the production of dry matter. PPFD can influence the RUE of plants by affecting the photosynthetic rate [10], stomatal behavior [11,12], photosynthate distribution [13,14], and chlorophyll content [15,16]. RUE is the highest under a PPFD of 200 μmol m^−2^ s^−1^ in lettuce and under a PPFD of 250 μmol m^−2^ s^−1^ in basil [17]. The optimal PPFD for high RUE among crops is different.

Light quality also influences the RUE of plants. McCree [18] reported that the relative quantum yield of photosynthesis was the highest at red wavelengths (600–700 nm). In addition, tomatoes (*Solanum lycopersicum* L. cv. Early Girl) grown in monochromatic red light produce more dry biomass than those grown in combinations of red and blue lights [19]. However, some studies in lettuce [20,21,22], cucumber [23], wheat [24], and rice [25] have indicated that plants have more biomass or higher photosynthetic rate under a combination of red and blue lights compared with monochromatic red light. Moreover, the effect of light quality on stomatal conductance in tomatoes varies with the genotype [26]. Such differences suggest that the effects of red and blue lights on plant biomass production and RUE are highly species- and cultivar-specific [19,27].

In addition to red and blue lights, white light has been used widely in research on lettuce [28,29,30], tomato [31], wheat [32,33], and vanilla [34] in the last 10 years, especially in the last 5 years, because of several advantages: first, white LED packages are now ~20% of the cost of red LEDs, which has contributed to the increase in the fraction of white LEDs to more than 60% in some horticultural fixtures [35]. Second, compared with red and blue lights, white light creates a more pleasant working environment and enables an easier visual assessment of plant health [30,36]. Lastly, white light contains useful wide wavebands of spectra other than red and blue lights, such as green light, which can increase photosynthesis and carbon assimilation in the lower parts of leaves [37,38]. Since white LEDs are widely used in PFALs, in this study, white light and combinations of red and blue lights were used to determine the suitable light quality for improving the RUE.

It is crucial to develop a strategy for controlling light conditions of dwarf tomatoes during the vegetative and reproductive growth stages. The growth of tomato plants is fast during the vegetative growth stage before anthesis [39]. The number of leaves of determinate tomatoes (i.e., Micro-Tom) is fixed during the vegetative growth stage; therefore, it is necessary to improve the leaf optical properties and photosynthetic capacity. In addition, light intensity and quality can affect the flowering of tomatoes, which is the principal determinant in yield [40,41]. Good growth at the vegetative stage is a foundation to further increase yield in the reproductive stage, and enhancing vegetative growth while maintaining a high RUE is the key approach for producing more tomatoes under limited energy. Therefore, we performed two experiments in this study (Experiments 1 and 2) to investigate the effects of PPFD and light quality on plant growth and RUE of dwarf tomatoes and to determine the suitable PPFD and light quality for improving the RUE at the vegetative growth stage.

## 2. Results

### 2.1. Experiment 1: Photosynthetic Photon Flux Density (PPFD) Effect

#### 2.1.1. Growth Characteristics

The total fresh and dry weights, leaf area (LA), and specific leaf area (SLA) were significantly affected by PPFD (Table 1). Seedlings under 700 μmol m^−2^ s^−1^ PPFD had the highest LA, and fresh and dry weights. SLA of plants was significantly higher under 300 μmol m^−2^ s^−1^ of PPFD compared with that under 500 and 700 μmol m^−2^ s^−1^. However, PPFD had no significant effect on the stem length. Furthermore, no significant differences were found in leaf number and dry matter ratio among the treatments.

#### 2.1.2. Light Interception and Radiation-Use Efficiency (RUE)

The increase in the projected leaf area (PLA) was similar among the three treatments (Figure S1). The daily averages of the intercepted PPFD proportions of the canopy in W300, W500, and W700 were 0.89, 0.88, and 0.87, respectively (Table 2). The intercepted PPFD increased with time for all treatments (Figure S2a), and the intercepted PPFD proportions exceeded 0.90 from 7 days after treatment (DAT) (Figure S2b). The dry mass increment (ΔW) from 3 to 9 DAT in W300, W500, and W700 were 0.34, 0.56, and 0.62 g, respectively (Table 1). RUE (Table 2) and photosynthetic light-use efficiency (pLUE) (Table 3) increased with the decrease in PPFD and were the highest for W300 among the three treatments. The intercepted PPFD proportions exceeded 0.90 when the leaf area index (LAI) was greater than three (Figure S3).

#### 2.1.3. Leaf Optical Properties and Chlorophyll Concentration

**Figure 1:**
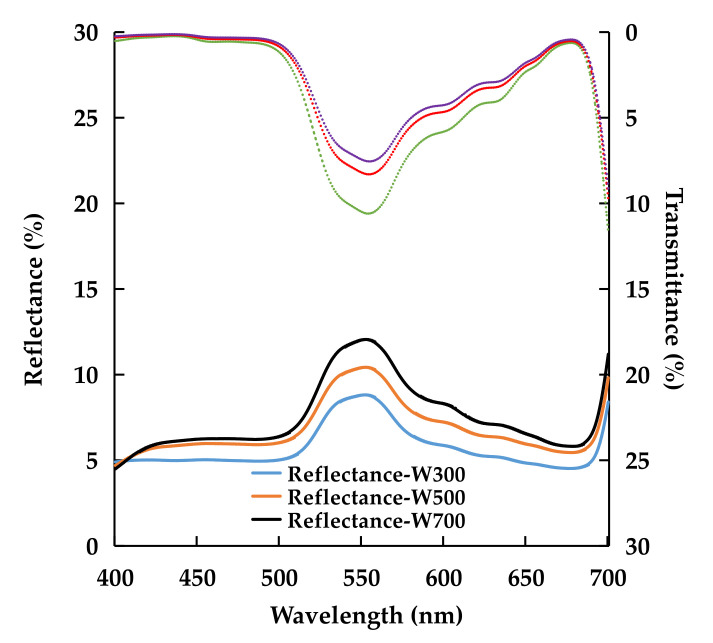
Effects of PPFD on the spectra of reflectance and transmittance of leaves in ‘Micro-Tom’ 10 DAT in Experiment 1. The range of measured light spectrum was 400–700 nm. W300, W500, and W700 denote 300, 500, and 700 μmol m^−2^ s^−1^ PPFD, respectively. Each value represents the average of the values of eight plants.

Top leaves under higher PPFDs (W500 and W700) reflected significantly more photosynthetically active radiation (PAR) than those in W300 (Table 4 and Figure 1). The transmittance of leaves under green light (500–599 nm) in W300 was significantly higher than that in W700 (Table 4). PPFD mostly affected the optical characteristics of the leaves in green light (Figure 1).

Chlorophyll concentration decreased as PPFD increased (Table 5). Chlorophyll concentration was the lowest in W700; moreover, the concentrations of both chlorophyll a and b decreased with the increase in PPFD. PPFD had no significant effect on chlorophyll a/b ratio.

#### 2.1.4. Net Photosynthetic Rate (Pn) and Light Response

**Figure 2:**
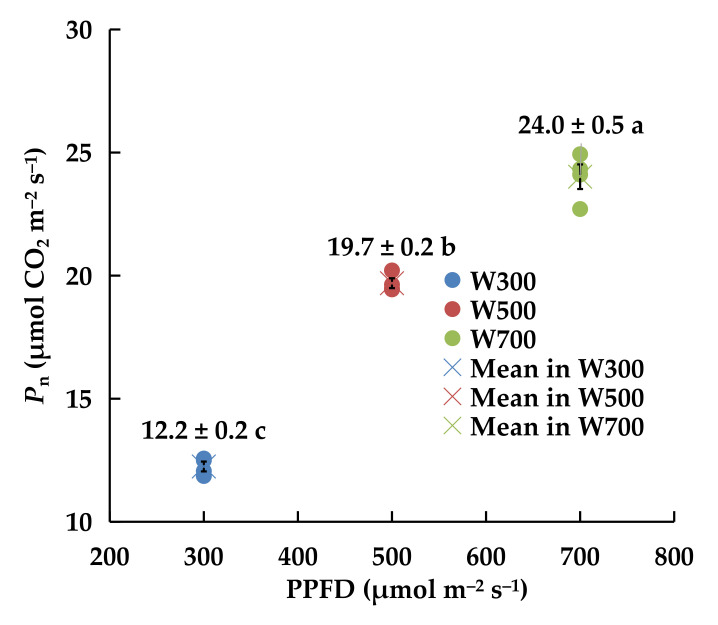
Effect of PPFD on net photosynthetic rate (Pn) of leaves in ‘Micro-Tom’ 10 DAT in Experiment 1. Four plants were measured for each PPFD. Solid point denotes the measured value for one plant. X-mark represents the average Pn of four plants in each treatment. Error bars represent ± standard error. Different letters indicate significant differences among the treatments based on Tukey−Kramer’s test at *p* < 0.05 (*n* = 4). W300, W500, and W700 denote 300, 500, and 700 μmol m^−2^ s^−1^ PPFD, respectively.

Pn differed significantly among the leaves of seedlings grown under different PPFD treatments (Figure 2). Increasing the PPFD from 300 to 700 μmol m^−2^ s^−1^ resulted in a significant increase in Pn from 12.2 to 24.0 µmol CO_2_ m^−2^ s^−1^, with the highest Pn obtained at a PPFD of 700 μmol m^−2^ s^−1^.

**Figure 3:**
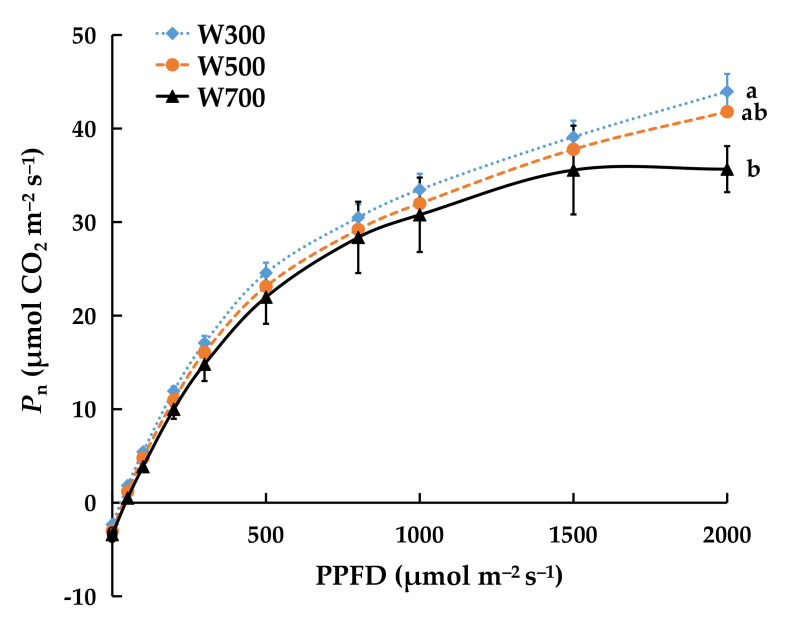
Light response curve of net leaf photosynthetic rate in ‘Micro-Tom’ 11 DAT in Experiment 1. The Pn to PPFD was determined on the third leaf (counted from top, fully expanded, and unshaded leaf). Each value represents the average of three plants. Error bars represent ± standard error. Different letters indicate significant differences among the treatments based on Tukey−Kramer’s test at *p* < 0.05. W300, W500, and W700 denote PPFDs of 300, 500, and 700 μmol m^−2^ s^−1^, respectively.

The Pn of the third leaf increased rapidly as PPFD increased to 500 μmol m^−2^ s^−1^ and then increased slowly to a maximum in all treatments (Figure 3). The Pn measured at PPFDs of 50, 100, and 2000 μmol m^−2^ s^−1^ in W700 were significantly lower than those in W300.

### 2.2. Experiment 2: Light Quality Effect

#### 2.2.1. Growth Characteristics

Light quality had a significant impact on the stem length, dry weight, dry matter ratio, and SLA (Table 6). The stem length under R3B1 was significantly lower than under W and R9B1. The dry weight and dry matter ratio under R9B1 were significantly higher than those under R3B1. Plants under R9B1 had the lowest SLA. There were no significant differences in leaf number, fresh weight, and LA among the three treatments.

#### 2.2.2. Light Interception and RUE

**Figure 4:**
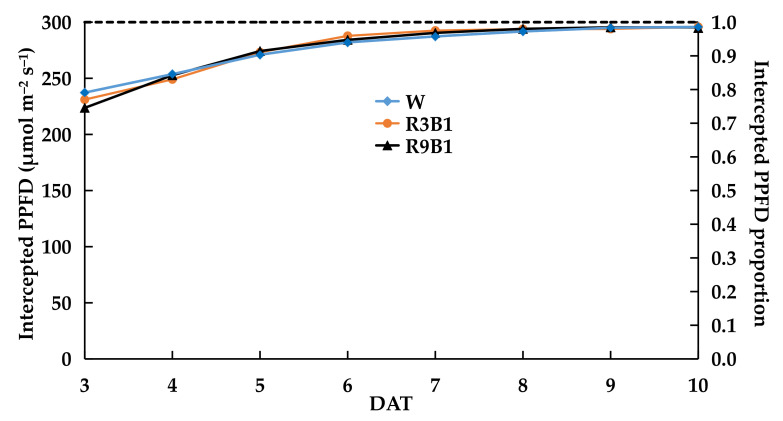
Effects of light quality on daily average of intercepted PPFD and proportion of the canopy in ‘Micro-Tom’ in Experiment 2. The intercepted PPFD of the canopy was calculated as the difference between the average PPFD on the top and bottom of the canopy. The intercepted PPFD proportion was calculated as the ratio of the intercepted PPFD to the average PPFD above the top of the canopy. The PPFD of the three treatments was set at 300 μmol m^−2^ s^−1^. W: white light; R3B1: red/blue ratio = 3; R9B1: red/blue ratio = 9.

The PLA of plants under R3B1 was the lowest from 8 to 10 DAT among the three treatments (Figure S4). The daily average of intercepted PPFD proportions of the canopy in W, R3B1, and R9B1 were 0.92 (Table 7). The intercepted PPFD increased with time for all treatments (Figure 4), and the intercepted PPFD proportions exceeded 0.90 from 6 DAT. The dry mass increments (ΔW) in W, R3B1, and R9B1 were 0.50, 0.46, and 0.56 g, respectively, from 3 to 10 DAT. The RUE of plants under R9B1 was the highest among the three treatments, being 1.18-fold higher than that under R3B1 (Table 7). Nevertheless, the RUE in R3B1 was almost the same as that in W. The lowest fraction of blue light (R9B1) led to the highest pLUE (Table 8). The intercepted PPFD proportions exceeded 0.90 when LAI was greater than three (Figure S5).

#### 2.2.3. Leaf Optical Properties and Chlorophyll Concentration

The leaves could reflect more green light under R9B1 than under W in Experiment 2 (Table 9 and Figure S6). The reflectance of the leaves was the lowest under W. There were no significant differences in the transmittance among the three treatments (Figure S6).

The concentration of chlorophyll decreased significantly with an increase in the R/B light ratio (Table 10). The chlorophyll concentration under W was significantly higher than that under R9B1. However, light quality had no significant effect on the chlorophyll a/b ratio.

#### 2.2.4. Pn and Light Response

**Figure 5:**
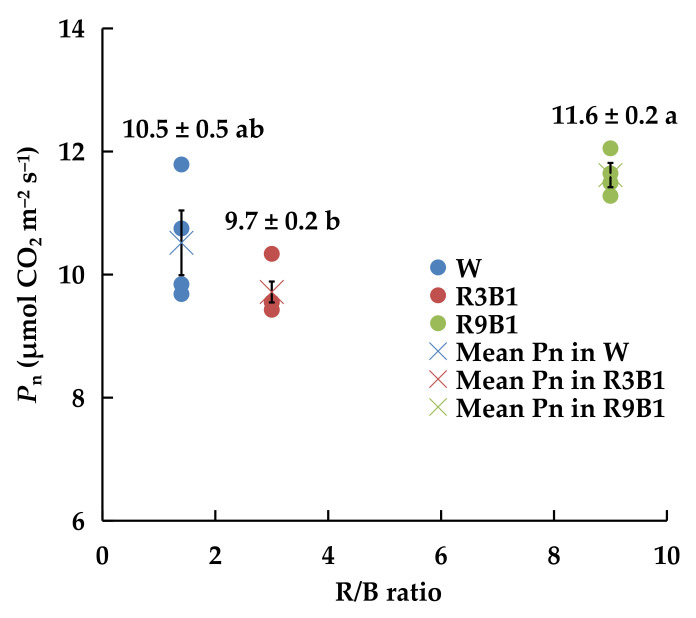
Effect of light quality on Pn of leaves in ‘Micro-Tom’ 10 DAT in Experiment 2. Four plants were measured for each PPFD. Solid point denotes the measured value for one plant. X-mark represents the average Pn of four plants in each treatment. Error bars represent ± standard error. Different letters indicate significant differences among the treatments based on Tukey−Kramer’s test at *p* < 0.05 (*n* = 4). W: white light; R3B1: red/blue ratio = 3; R9B1: red/blue ratio = 9.

Pn differed significantly among the leaves grown under different proportions of R and B lights (Figure 5). The Pn of leaves under R9B1 (11.6 µmol CO_2_ m^−2^ s^−1^) was significantly higher than that under R3B1 (9.7 µmol CO_2_ m^−2^ s^−1^).

The Pn of the third leaf increased rapidly as PPFD increased to 500 μmol m^−2^ s^−1^ and then increased slowly to a maximum in all treatments (Figure S7). At a PPFD of 2000 μmol m^−2^ s^−1^, Pn under W was significantly lower than that under R9B1 and R3B1. However, there were no significant differences in Pn at other values of PPFD.

## 3. Discussion

### 3.1. Experiment 1: PPFD Effect

Experiment 1 demonstrated that PPFD had significant effects on total fresh and dry weights, LA, and SLA (Table 1). Generally, plants have long stems, resulting in tall plants, to promote light capture under shade conditions [42,43]. Some studies conducted in PFALs or growth chambers with artificial light indicated that stem elongation decreased with an increase in PPFD [44,45,46]. However, other studies have reported that high PPFD results in long stems, thereby increasing the plant height [47,48]. In the case of tomatoes, the plant height of the cherry tomato seedlings (*Solanum lycopersicum* cv. Mill qianxi) decreased when PPFD increased from 50 to 500 μmol m^−2^ s^−1^ [49]. A similar result was demonstrated in a study by Matsuda et al. [50], in which the stem length of the tomato cultivar ‘Momotaro Fight’ was significantly longer at a PPFD of 150 μmol m^−2^ s^−1^ than that of 300 μmol m^−2^ s^−1^. Another study by He et al. [14] used the ‘Mill qianxi’ tomato cultivar and found that plant height increased when PPFD was increased from 50 to 300 μmol m^−2^ s^−1^ but decreased when PPFD was further increased to 550 μmol m^−2^ s^−1^. The stem lengths of different species and cultivars might respond differently to PPFD under different experimental conditions. In Experiment 1, there was no significant difference in the stem length of plants under different PPFD treatments, probably because the cultivar ‘Micro-Tom’ is a dwarf tomato. Experiment 1 verified that higher PPFD leads to lower LA (Figure S8) and SLA [49] (PPFD: 50–550 μmol m^−2^ s^−1^), and higher fresh and dry weights [51] (PPFD: 50–600 μmol m^−2^ s^−1^) of plants under LED light (Table 1).

The ratio of PAR above the top of the canopy in W300, W500, and W700 was 3:5:7. The ratio of average PAR received by one plant in the three treatments was 3.0:4.9:6.6 (Table 2). However, the ratio of dry mass produced by one plant during the period was close to 3.0:4.8:5.5, which differed from the 3.0:4.9:6.6 ratio. The plants under W700 intercepted a lower proportion of PPFD and produced less dry mass per mole PAR photons than those under W300 and W500 during the 6-day experimental period. In addition, PPFD increased Pn (Figure 2) but reduced pLUE (Table 3). Jayalath and van Iersel [10] also reported that a high PPFD decreased the RUE by reducing the quantum yield of photosystem II. In Experiment 1, the RUE of plants in W300 was the highest among the three treatments. Therefore, 300 μmol m^−2^ s^−1^ was selected as the proper value of PPFD above the top of the canopy for ‘Micro-Tom’ cultivation at the vegetative growth stage to enhance the RUE.

In natural conditions, light is one of the limiting resources, and plants grown under low PPFD must adapt to capture light efficiently [52]. In contrast, leaves under high PPFD conditions have thicker cuticles and lower SLA and chlorophyll concentrations than those grown under low PPFD conditions [53,54]. These characteristics increase the reflectance of leaves [54,55], which may explain the higher reflectance and lower transmittance (lower absorptance) of leaves under high PPFD than those under low PPFD (Table 4 and Figure 1). In addition, lower leaves shared more light because of the significantly low reflectance and absorptance of the top-layer leaves at low PPFD, and might have a more uniform vertical PPFD distribution, which could contribute to the canopy RUE [56]. He et al. [14] reported that leaf chlorophyll concentration in tomatoes increased with a decrease in PPFD within a certain range (300–550 μmol m^−2^ s^−1^). To capture more light under a low PPFD, leaves must distribute more dry mass to produce more chlorophyll. Under excessive or long-term high PPFD, chlorophyll could be damaged or destroyed, resulting in a low chlorophyll concentration (Table 5).

Usually, leaves have lower photosynthetic rates at high PPFD but higher photosynthetic rates at low PPFD [57,58,59]. Our results showed that the photosynthetic capacity of the leaves (the maximum rate at which leaves can fix carbon during photosynthesis) was higher under W300 than under W700, which indicated that the photosynthetic capacity or potential was attenuated under a PPFD of 700 µmol m^−2^ s^−1^ (Figure 3). Long-term exposure of plants to excessive light leads to the production of large amounts of reactive oxygen species, thereby superseding the capacity of antioxidant systems and resulting in irreversible photooxidative damage to the chloroplast and cells, thus inhibiting photosynthesis [60,61]. Hence, the PPFD of 700 µmol m^−2^ s^−1^ might have been too high for the efficient production of ‘Micro-Tom’.

### 3.2. Experiment 2: Light Quality Effect

Light quality can alter plant morphology by photoreceptors and signal transduction systems [27,62]. Light quality affected the stem length and total dry weight in Experiment 2 (Table 6). In contrast to blue light, red light is known to increase the length of hypocotyl and plant height [41,63,64]. Our findings were consistent with this observation in Experiment 2, which showed that the stem length of seedlings grown under R9B1 light was the highest (Table 6). In contrast, the plants grown under R3B1, which had a higher percentage of blue light, produced significantly less dry mass than those grown under R9B1. This result was consistent with the finding that a high percentage of blue light can inhibit biomass production [65,66,67].

Similar to PPFD, light quality had little effect on the proportion of intercepted light 6 DAT (Figure 4). However, the ratio of dry biomass produced during the period was 0.50:0.46:0.56 (≈1.0:0.9:1.1). McCree [18] elucidated that the relative quantum efficiency of single leaves under blue light (400–500 nm) was 65–75% of that under red light (600–700 nm). In addition, a higher fraction of blue light led to lower Pn (Figure 5) and pLUE (Table 8), and this trend was similar to the changes in RUE (Table 7). This may explain the reason for the highest RUE of plants under the lowest fraction of blue light (R9B1). Therefore, R9B1 was the proper light quality for ‘Micro-Tom’ cultivation at the vegetative growth stage to increase the growth and RUE.

However, this does not necessarily mean that a greater proportion of red light leads to a higher RUE. In Experiment 2, R3B1 had a higher proportion of red light compared with W. In contrast, RUE was higher under W than under R3B1. McCree [18] found that the relative quantum efficiency of green light (500–600 nm) was not inferior to that of blue light. In addition, green light can penetrate deeper into the canopy than red and blue light and improve the photosynthesis of single leaves and whole plants [37,68], which might be the reason for the higher RUE of plants under the highest fraction of green light (W) than under R3B1. Dong et al. [33] reported a similar case in wheat plants and found that both photosynthetic rate and stomatal conductance of leaves under monochromatic red light and a combination of red and blue lights were lower than those under white light.

Red light does not promote chlorophyll synthesis but sometimes inhibits it [21,22,69]. This was corroborated by our results of Experiment 2, in which chlorophyll concentration was the highest under the lowest red light fraction in W (Table 10). The highest chlorophyll concentration under W caused the lowest reflectance of leaves under green light (500–599 nm) among the three treatments (Table 9).

The effects of PPFD and light quality on dwarf tomatoes were different. Light quality had a significant effect on stem length, unlike PPFD. PPFD significantly affected LA, SLA, and leaf optical properties and changed the intercepted light proportion. Light quality did not significantly affect LA, SLA, light absorptance of leaves, or the intercepted light proportion. However, only one cultivar of dwarf tomato was used in this study, and the effects of PPFD and light quality on the growth and RUE may differ among tomato cultivars. Therefore, more tomato cultivars with efficient environment control strategies may be studied in the future to popularize efficient tomato production in PFALs. Moreover, 200–300 μmol m^−2^ s^−1^ PPFD and light quality with an RB ratio greater than three are used in a lettuce-production PFAL which is the most popular PFAL type [17,70]. Therefore, it is easy to efficiently produce dwarf tomatoes using a lettuce-production PFAL after simple modification.

## 4. Materials and Methods

### 4.1. Plant Material and Growth Conditions

A dwarf tomato cultivar, ‘Micro-Tom’ (*Solanum lycopersicum* L.), was used for the experiment. Tomato seeds were sown in a urethane sponge (M-urethane, M Hydroponics Laboratory Co. Inc., Aichi, Japan) and maintained at 25 °C for 3 days in the dark. Seeds germinated on the 3rd day, and then seedlings were cultivated under white LED lamps (LDL40S-N19/21, Panasonic Corporation, Osaka, Japan) in a room with a controlled environment at Matsudo campus, Chiba University. The following conditions were maintained—the PPFD was set to 200 μmol m^−2^ s^−1^ at the canopy level using a quantum sensor (LI-190, LI-COR Inc., Lincoln, NE, USA) and air temperature of 25/20 °C (light/dark period) with 70% relative humidity, 1000 μmol mol^−1^ CO_2_ concentration, and 16 h/8 h (light/dark) photoperiod were maintained. All plants were cultivated using 1/2 OAT house A nutrient (OAT Agrio Co., Ltd., Tokyo, Japan) from 7 days after germination. pH and electrical conductivity of the nutrition solution were set at 6.3 and 1.3 dS m^−1^, respectively. The nutrition solution was renewed every 4 days. Seedlings were grown under these conditions until the third true leaf (from the bottom) fully expanded, which occurred 24 days after sowing, after which the seedlings were transplanted into the treatment area.

### 4.2. Experimental Conditions

#### 4.2.1. Experiment 1: PPFD Effect

**Figure 6:**
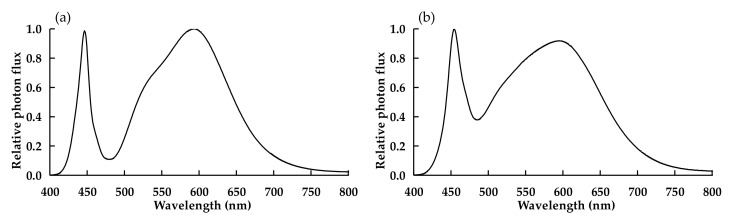

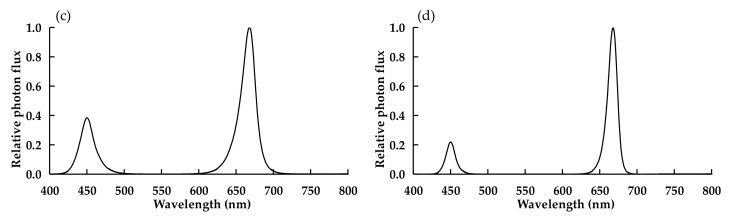
Spectral photon flux distributions of white LED lamp (customized lamp) in Experiment 1 (**a**), white LED lamp (LDL40S-N19/21) in Experiment 2 (**b**), as well as red and blue LED lamps in R3B1 (**c**) and R9B1 (**d**) in Experiment 2. R3B1 and R9B1 represent photon flux ratios (red to blue light) of 3:1 and 9:1, respectively. The peak wavelengths of the white lamp were 446 nm and 592 nm in Experiment 1 and 454 nm and 593 nm in Experiment 2. The peak wavelengths of red and blue light were 667 and 450 nm, respectively, in Experiment 2. The maximum value of photon flux was converted to 1.0.

Ninety-six uniform seedlings were equally divided and inserted into three polystyrene foam boards placed over 18.6 L containers (600 mm × 300 mm × 141 mm; SANKO Co. Ltd., Tokyo, Japan). Each container was placed under one of the three different PPFDs in a growth chamber with white LED lamps (customized lamp, color temperature: 4000 K; Showa Denko K. K., Tokyo, Japan). The different light treatments were: W300 (PPFD: 300 μmol m^−2^ s^−1^, daily light integral (DLI): 17.28 mol m^−2^ d^−1^), W500 (PPFD: 500 μmol m^−2^ s^−1^, DLI: 28.80 mol m^−2^ d^−1^), and W700 (PPFD: 700 μmol m^−2^ s^−1^, DLI: 40.32 mol m^−2^ d^−1^). The above PPFDs were the average values above the top of the canopies and were chosen based on a previous study (data not shown). The environmental conditions, except for light conditions, were the same as those before transplanting. The spectral photon flux distribution of the white LED lamp was measured using a spectroradiometer (USR-45DA, USHIO Inc., Tokyo, Japan), as shown in Figure 6. The fractions of blue, green, and red wavelengths, as well as red/blue ratio, were calculated (Table 11). The planting density of seedlings was 476.2 plant m^−2^. The side shoots of all plants were pruned when visible. The first flowers of half of the plants bloomed 10 DAT.

#### 4.2.2. Experiment 2: Light Quality Effect

Ninety-six uniform seedlings were equally divided into three containers, as in Experiment 1. The three containers were placed in a growth chamber with three different light quality lamps. The spectral photon flux distributions of the white LED lamp (LDL40S-N19/21, Panasonic Corporation, Osaka, Japan) and RB LED lamps (CIVILIGHT, DPT2RB120Q33 40 type, Showa Denko K.K., Tokyo, Japan) are shown in Figure 6. The fractions of blue, green, and red wavelength ranges of the lamps are listed in Table 11. Different light quality treatments—W (white light) and a mixture of red (R) and blue (B) lights, R3B1 (R:B = 3:1) and R9B1 (R:B = 9:1)—were administered. The PPFD of the three treatments was set at 300 μmol m^−2^ s^−1^ based on the results of Experiment 1. The environmental conditions, except for the light conditions, were the same as in Experiment 1.

### 4.3. Growth Measurement

Growth parameters were measured every 2–5 days from 0 to 9 DAT in Experiment 1, to 10 DAT in Experiment 2, respectively. The growth parameters measurement in both experiments were performed with two replicates. Stem length was measured from the main stem base to the top of the stem with a ruler. ‘Micro-Tom’ is one kind of determinate tomato. There is no new leaf on the main stem after the first truss. The number of leaves on the main stem, which is also the leaf number until the first truss, was recorded. Eight and nine plants were used in each treatment of Experiments 1 and 2 in two replicates and were sampled destructively for biomass analysis as well as leaf area measurement at the end of the experiments, respectively. Fresh and dry weights of plant organs (leaves, stems, and roots) were determined. Plant organs were dried for 72 h at 80 °C in a convection oven. LA (cm^2^) was measured using a leaf area meter (LI-3000C, LI-COR Inc., Lincoln, NE, USA). LAI was defined as the ratio of LA to cultivation area (cm^2^). SLA (cm^2^ g^−1^) was calculated by dividing LA (cm^2^) by the fresh leaf weight (g).

### 4.4. RUE of Canopy

Understanding the basic determinants of RUE is vital for enhancing the productivity and energy use efficiency of dwarf tomatoes in PFALs. It is necessary to carefully use the RUE definitions in PFALs. Some studies used g mol^−1^ as the unit of RUE and calculated it based on the sum of PAR on the cultivation area or irradiation by lamps, which focused on the production efficiency rather than the physiological mechanisms [73,74,75]. Recently, a few studies have defined RUE based on the cumulative incident radiation on the PLA [10,76,77]. Certainly, the intercepted radiation is nearly equal to the incident radiation when the LAI is sufficiently large, or almost all the incident radiation is absorbed by the plant. When the LAI is small, a part of the incident radiation is irradiated on the cultivation surface and is not absorbed by the plant. Therefore, we chose the latter method in this study.

RUE (g mol^−1^) is defined as the proportion of the accumulated total dry mass to the integrated PPFD received by the plant during a given period.

(1) $$\mathrm{RUE}=\frac{\Delta W}{I_{\mathrm{PPFD}}}$$

where ΔW (g) is the accumulated total dry mass during a period, and I_PPFD_ (mol) is the integrated PPFD received by the plant during a given period (from 3 to 9 DAT in Experiment 1 and from 3 to 10 DAT in Experiment 2, respectively).

ΔW (g) during a given period (t_1_ − t_0_) is defined as follows:

(2) $${\Delta W=W}_{t_{1}}-W_{t_{0}}\;(t_{0}\;<\;t_{1})$$

where W_t0_ (g) and W_t1_ (g) are the total dry mass of the plant on the first day (t_0_) and the last day (t_1_) of a given period, respectively.

The I_PPFD_ (mol) during a given period (t_1_ − t_0_) was calculated as follows:

(3) $$I_{\mathrm{PPFD}}=a\times \sum_{t=t_{0}}^{t_{1}}[\mathrm{PLA}\left(t\right)\times ({\mathrm{PPFD}}_{T\;}-\;\mathrm{PPFD}(t))]\;(t_{0}\;\leq \;t\;\leq \;t_{1})$$

where ‘a’ is the conversion factor for the light period of 1 day—5.76 × 10^4^ s (16 h × 3600 s h^−1^). PLA(t) is the PLA (m^2^) of the plant on day t. PPFD_T_ is the PPFD at the top of the canopy (mol m^−2^ s^−1^), and it is specific for each treatment. PPFD(t) is the PPFD (mol m^−2^ s^−1^) at the bottom of the canopy on day t.

The PPFDs above the top of the canopies were measured every day using a quantum sensor (LI-190, LI-COR Inc., Lincoln, NE, USA) and GaAsp photodiodes (G1118, Hamamatsu Photonics K. K., Shizuoka, Japan), and maintained at 300, 500, and 700 μmol m^−2^ s^−1^ in W300, W500, and W700 in Experiment 1, respectively. PPFDs of 69–86 evenly distributed points on the bottom of the canopy and on the surface of the polystyrene foam board were measured with the quantum sensor from 3 to 9 DAT. PPFDs of 40–50 evenly distributed points above the top of the canopy were measured with the GaAsp photodiodes. In contrast, PPFDs above the top of canopies in Experiment 2 were maintained at 300 μmol m^−2^ s^−1^ in all cases. PPFDs of 43–80 measurement points on the canopy bottom were measured with the quantum sensor every day from 3 to 10 DAT. The intercepted PPFD of the canopy was calculated as the difference between the average PPFD above the top and on the bottom of the canopy. The intercepted PPFD proportion was calculated as the ratio of the intercepted PPFD to the average PPFD above the top of the canopy.

Photographs of the canopy were taken from the top every day, and PLA per plant [78] was determined from the photographs using a free imaging software (LIA 32 ver. 0.378, Yamamoto) from 1 to 9 DAT in Experiment 1 and from 2 to 10 DAT in Experiment 2.

The pLUE was defined as RUE directly at the leaf level in some studies [79,80] and can reflect the RUE of the canopy in this study. The pLUE (expressed in mmol CO_2_/mol photon) was calculated as the ratio of the Pn to PPFD.

### 4.5. Leaf Optical Properties and Chlorophyll Concentration

The reflection and transmission spectra of the third leaf (fully expanded and unshaded leaf) from the top of the plant were measured using a spectrophotometer (V-750, JASCO Corporation, Tokyo, Japan) with an integrating sphere unit (ISV-922, JASCO Corporation, Tokyo, Japan) at 10 DAT. The range of the measured light spectrum was 400–700 nm. Eight and four plants in each treatment in Experiments 1 and 2 were sampled, respectively. Absorptance was calculated for each wavelength as follows:

Absorptance = 100% − (Reflectance + Transmittance)(4)

Chlorophyll pigment was extracted from the third leaf from the top of the plant with N,N-dimethylformamide at 10 DAT, according to the protocol described by Porra et al. [81]. For chlorophyll concentration analysis, four leaves from four plants in each treatment were sampled. The chlorophyll concentration was determined on a dry weight basis by measuring the absorbance of the leaf extracts at 663.8, 646.8, and 750.0 nm using an ultraviolet-visible spectrophotometer (V-750, JASCO Corporation, Tokyo, Japan).

### 4.6. Gas Exchange

#### 4.6.1. Leaf Pn

The Pn was determined for the third leaf of four randomly selected plants in each treatment at 10 DAT using a portable photosynthesis measurement system (LI-6400XT, LI-COR Inc., Lincoln, NE, USA), equipped with a transparent cuvette under the following environmental conditions: 25 ± 1 °C leaf temperature, 65–70% relative humidity, and 1000 μmol mol^−1^ CO_2_ concentration. The leaves were clamped into the cuvette until the Pn and stomatal conductance were stable at every measurement. The flow rate of air through the system was set to 500 μmol s^−1^.

#### 4.6.2. Light Response Curves

The response of Pn to PPFD was also determined on the third leaf in each treatment using the LI-6400XT photosynthesis measurement system, equipped with a 6400-02B LED light source (90% red light with peak at 665 nm, and 10% blue light with peak at 470 nm) in a leaf chamber. The leaves were clamped into the cuvette at 1000 μmol m^−2^ s^−1^ PPFD until the Pn and stomatal conductance were stable. The following PPFD gradient was set at the leaf surface—2000, 1500, 1000, 800, 500, 300, 200, 100, 50, and 0 μmol m^−2^ s^−1^. Leaf temperature, relative humidity, and CO_2_ concentration were set at 25 ± 1 °C, 65–70%, and 1000 μmol mol^−1^, respectively. The flow rate of air through the system was set to 500 μmol s^−1^.

### 4.7. Statistical Analysis

Data were statistically evaluated by one-way analysis of variance (ANOVA) using the SPSS program for Windows (Version 24.0; SPSS Inc., Chicago, IL, USA). To investigate significant differences among the treatments, the mean value of the measured data was compared using the Tukey−Kramer test at *p* < 0.05.

## 5. Conclusions

In this study, we evaluated the effects of PPFD and light quality on the growth and RUE in the dwarf tomato cultivar ‘Micro-Tom’. The results demonstrated that higher PPFD caused higher dry mass production and lower SLA, but hardly affected the stem length. In addition, higher PPFD increased the Pn of individual leaves but decreased the RUE of the canopy. This was probably because the pLUE decreased with increasing PPFD. A PPFD of 700 μmol m^−2^ s^−1^ was excessive and decreased the photosynthetic capacity and chlorophyll concentration. Moreover, a high proportion of blue light inhibited dry mass production with the same intercepted light because leaves under a higher blue light proportion had lower Pn and pLUE. In conclusion, 300 μmol m^−2^ s^−1^ PPFD and R9B1 are recommended for ‘Micro-Tom’ cultivation at the vegetative growth stage to improve the RUE. The results of our study would be helpful in efficient tomato production in PFALs. Further study is necessary to determine a proper light condition for the reproductive growth stage to produce fruits by improving the RUE.

## Figures and Tables

**Table 1:** Effect of photosynthetic photon flux density (PPFD) on the growth of ‘Micro-Tom’ 9 days after treatment (DAT) in Experiment 1. W300, W500, and W700 denote PPFD of 300, 500, and 700 μmol m^−2^ s^−1^, respectively. Each value represents the mean ± standard error. Different letters in a column indicate significant differences among the treatments based on the Tukey−Kramer’s test (*n* = 8) at *p* < 0.05.

Treatment	Stem Length (cm)	Leaf Number	Total Fresh Weight (g)	Total Dry Weight (g)	Dry Matter Ratio (%)	Leaf Area (cm^2^)	Specific Leaf Area (cm^2^ g^−1^)
0 DAT	2.4 ± 0.1	3.5 ± 0.2	0.50 ± 0.03	0.05 ± 0.00	9.81 ± 0.20		
W300	3.4 ± 0.2	6.6 ± 0.2	5.69 ± 0.54 c	0.50 ± 0.04 c	8.81 ± 0.15	73.46 ± 4.18 b	257.60 ± 14.92 a
W500	3.8 ± 0.1	6.6 ± 0.2	7.71 ± 0.60 b	0.73 ± 0.06 b	9.55 ± 0.15	83.66 ± 2.33 b	199.68 ± 20.14 b
W700	3.8 ± 0.2	6.4 ± 0.2	9.55 ± 0.33 a	0.92 ± 0.05 a	9.57 ± 0.30	95.50 ± 2.01 a	167.96 ± 9.10 b

**Table 2:** Effects of PPFD on light interception, biomass production, and radiation-use efficiency (RUE) of ‘Micro-Tom’ during the 6-day experimental period in Experiment 1. ΔW (g) is the accumulated total dry mass during the experimental period. RUE is defined as the ratio of the accumulated total dry mass to integrated PPFD received by the plant during the period. W300, W500, and W700 denote 300, 500, and 700 μmol m^−2^ s^−1^ PPFD, respectively. Each value of ΔW represents the average of four plants.

Treatment	Integrated PPFD Received by the Plant during the Period (mol)	Daily Average Intercepted PPFD Proportion during the Period	ΔW (g)	RUE (g mol^−1^)
W300	0.30	0.89	0.34	1.15
W500	0.49	0.88	0.56	1.14
W700	0.66	0.87	0.62	0.94

**Table 3:** Effect of PPFD on the photosynthetic light-use efficiency (pLUE) of leaves in ‘Micro-Tom’ 10 DAT in Experiment 1. pLUE is the ratio of net photosynthetic rate to PPFD (mmol CO_2_/mol photon). Each value represents the mean ± standard error. Different letters indicate significant differences among the treatments based on Tukey−Kramer’s test at *p* < 0.05 (*n* = 4). W300, W500, and W700 denote 300, 500, and 700 μmol m^−2^ s^−1^ PPFD, respectively.

Treatment	pLUE (mmol CO_2_/mol Photon)
W300	40.82 ± 0.57 a
W500	39.38 ± 0.36 a
W700	34.31 ± 0.67 b

**Table 4:** Effects of PPFD on the transmittance and reflectance of leaves in ‘Micro-Tom’ at blue, green, and red wavelengths 10 DAT in Experiment 1. The range of measured light spectrum was 400–700 nm. Each value represents the mean ± standard error. Different letters in a column indicate significant differences among the treatments based on the Tukey−Kramer’s test at *p* < 0.05 (*n* = 8). W300, W500, and W700 denote 300, 500, and 700 μmol m^−2^ s^−1^ PPFD, respectively.

Treatment	Transmittance (%)			Reflectance (%)		
	400–499 nm (Blue)	500–599 nm (Green)	600–700 nm (Red)	400–499 nm (Blue)	500–599 nm (Green)	600–700 nm (Red)
W300	0.5 ± 0.1	7.3 ± 0.7 a	3.4 ± 0.4	5.0 ± 0.1b	7.1 ± 0.3 b	5.2 ± 0.1 b
W500	0.3 ± 0.1	5.6 ± 0.5 ab	2.7 ± 0.4	5.8 ± 0.1 a	8.5 ± 0.2 a	6.3 ± 0.1 a
W700	0.3 ± 0.0	5.1 ± 0.5 b	2.5 ± 0.3	6.0 ± 0.3 a	9.7 ± 0.5 a	6.9 ± 0.3 a

**Table 5:** Effect of PPFD on chlorophyll concentration (conc.) of leaves in ‘Micro-Tom’ 10 DAT in Experiment 1. DW (g) is dry weight. Each value represents the mean ± standard error. Different letters in a column indicate significant differences among the treatments based on Tukey−Kramer’s test at the *p* < 0.05 (*n* = 4). W300, W500, and W700 denote 300, 500, and 700 μmol m^−2^ s^−1^ PPFD, respectively.

Treatment	Chlorophyll a Conc. (mg g^−1^ DW)	Chlorophyll b Conc. (mg g^−1^ DW)	Chlorophyll a + b Conc. (mg g^−1^ DW)	Chlorophyll a/b
W300	2.07 ± 0.20 a	0.59 ± 0.04 a	2.66 ± 0.24 a	3.52 ± 0.21
W500	1.67 ± 0.06 ab	0.46 ± 0.02 b	2.13 ± 0.08 a	3.61 ± 0.07
W700	1.25 ± 0.02 b	0.34 ± 0.00 c	1.59 ± 0.02 b	3.66 ± 0.04

**Table 6:** Effect of light quality on the growth of ‘Micro-Tom’ 10 DAT in Experiment 2. Each value represents the mean ± standard error. Different letters in a column indicate significant differences among the treatments based on Tukey−Kramer’s test at *p* < 0.05 (*n* = 9). W: white light; R3B1: red/blue ratio = 3; R9B1: red/blue ratio = 9.

Treatment	Stem Length (cm)	Leaf Number	Total Fresh Weight (g)	Total Dry Weight (g)	Dry Matter Ratio (%)	Leaf Area (cm^2^)	Specific Leaf Area (cm^2^ g^−1^)
0 DAT	3.5 ± 0.3	3.4 ± 0.1	0.55 ± 0.03	0.06 ± 0.00	10.92 ± 0.41	-	-
W	4.7 ± 0.1 a	6.4 ± 0.2	7.93 ± 0.46	0.66 ± 0.04 ab	8.34 ± 0.12 b	130.60 ± 4.12	313.59 ± 6.91 a
R3B1	4.4 ± 0.0 b	6.4 ± 0.2	7.10 ± 0.39	0.59 ± 0.03 b	8.33 ± 0.21 b	115.13 ± 5.91	308.03 ± 6.07 a
R9B1	4.8 ± 0.1 a	6.0 ± 0.0	7.90 ± 0.27	0.71 ± 0.03 a	9.00 ± 0.52 a	130.36 ± 4.79	281.91 ± 4.51 b

**Table 7:** Effects of light quality on light interception, biomass production, and RUE of ‘Micro-Tom’ during the 7-day experimental period in Experiment 2. ΔW (g) is the total dry mass accumulated during the experimental period. Each value of ΔW represents the average of six plants. RUE (g mol^−1^) is defined as the ratio of the accumulated total dry mass to integrated PPFD received by the plant during the experimental period. W: white light; R3B1: red/blue ratio = 3; R9B1: red/blue ratio = 9.

Treatment	Integrated PPFD Received by the Plant during the Period (mol)	Daily Average Intercepted PPFD Proportion during the Period	ΔW (g)	RUE (g mol^−1^)
W	0.43	0.92	0.50	1.15
R3B1	0.40	0.92	0.46	1.13
R9B1	0.42	0.92	0.56	1.36

**Table 8:** Effect of light quality on the pLUE of leaves in ‘Micro-Tom’ 10 DAT in Experiment 2. pLUE is the ratio of net photosynthetic rate to PPFD and expressed in mmol CO_2_/mol photon. Each value represents the mean ± standard error. Different letters indicate significant differences among the treatments based on Tukey−Kramer’s test at the *p* < 0.05 (*n* = 4). W: white light; R3B1: red/blue ratio = 3; R9B1: red/blue ratio = 9.

Treatment	pLUE (mmol CO_2_/mol Photon)
W	35.05 ± 1.62 ab
R3B1	32.39 ± 0.70 b
R9B1	38.72 ± 0.54 a

**Table 9:** Effect of light quality on the reflectance of leaves in ‘Micro-Tom’ at blue, green, and red wavelengths 10 DAT in Experiment 2. The range of the measured light spectrum was 400–700 nm. Each value represents the mean ± standard error. Different letters in a column indicate significant differences among the treatments based on Tukey−Kramer’s test at *p* < 0.05 (*n* = 4). The PPFD of three treatments was set at 300 μmol m^−2^ s^−1^. W: white light; R3B1: red/blue ratio = 3; R9B1: red/blue ratio = 9.

Treatment	Reflectance (%)		
	400–499 nm (Blue)	500–599 nm (Green)	600–700 nm (Red)
W	5.1 ± 0.3	7.4 ± 0.2 b	5.3 ± 0.1
R3B1	5.6 ± 0.1	7.8 ± 0.2 ab	5.7 ± 0.2
R9B1	5.3 ± 0.1	8.4 ± 0.3 a	5.7 ± 0.2

**Table 10:** Effect of light quality on chlorophyll concentration (conc.) of leaves in ‘Micro-Tom’ 10 DAT in Experiment 2. DW (g) is dry weight. Each value represents the mean ± standard error. Different letters in a column indicate significant differences among the treatments based on Tukey−Kramer’s test at *p* < 0.05 (*n* = 4). The PPFD of three treatments was set at 300 μmol m^−2^ s^−1^. W: white light; R3B1: red/blue ratio = 3; R9B1: red/blue ratio = 9.

Treatment	Chlorophyll a Conc. (mg g^−1^ DW)	Chlorophyll b Conc. (mg g^−1^ DW)	Chlorophyll a + b Conc. (mg g^−1^ DW)	Chlorophyll a/b
W	2.29 ± 0.12 a	0.73 ± 0.05 a	3.01 ± 0.15 a	3.15 ± 0.06
R3B1	1.96 ± 0.06 ab	0.61 ± 0.02 ab	2.57 ± 0.07 ab	3.22 ± 0.02
R9B1	1.69 ± 0.05 b	0.52 ± 0.02 b	2.21 ± 0.06 b	3.24 ± 0.04

**Table 11:** Spectral data for LED lamps at the wavelength of 400–700 nm. ‘%’ represents the ratios of blue, green, red, and far-red photon fluxes as a percentage of photon flux density. R/B ratio represents the photon flux ratio of red light to blue light. ‘W’ represents white LED lamps. R3B1 and R9B1 represent red-blue LED lamps with photon flux ratios of 3:1 and 9:1, respectively, of red to blue light. The phytochrome photostationary state (PSS) is calculated using spectral composition and intensity of light received by plants [71,72].

	W in Experiment 1	W in Experiment 2	R3B1	R9B1
% Blue (400–499 nm)	17.1	21.5	24.5	9.9
% Green (500–599 nm)	46.7	42.9	0.4	0.4
% Red (600–699 nm)	32.9	31.5	74.7	89.0
% Far-red (700–800 nm)	3.3	4.0	0.4	0.7
R/B ratio	1.9	1.5	3.0	9.0
PSS	0.85	0.84	0.87	0.88

## Data Availability

Data is contained within the article or Supplementary Materials.

## Supplementary Materials

The following are available online at https://www.mdpi.com/article/10.3390/plants11010121/s1, Figure S1: Effect of photosynthetic photon flux density (PPFD) on the projected leaf area (PLA) per plant of ‘Micro-Tom’ in Experiment 1; Figure S2: Effects of PPFD on the daily average of intercepted PPFD (a) and daily average of intercepted proportion (b) of the canopy in ‘Micro-Tom’ in Experiment 1; Figure S3: Relationships between leaf area index (LAI) and average PPFD on the bottom of the canopy and intercepted PPFD proportion under different PPFD in Experiment 1; Figure S4: Effect of light quality on the PLA of ‘Micro-Tom’ in Experiment 2; Figure S5: Relationships between LAI and average PPFD on the bottom of the canopy and intercepted PPFD proportion under different light qualities in Experiment 2; Figure S6: Effects of light quality on the reflectance and transmittance spectra of leaves in ‘Micro-Tom’ 10 DAT in Experiment 2; Figure S7: Light response curve of net leaf photosynthetic rate in ‘Micro-Tom’ 11 DAT in Experiment 2; Figure S8: ‘Micro-Tom’ seedlings grown under different PPFDs 10 DAT in Experiment 1.
