# Supplementary material for: Optimization of Photosynthetic Photon Flux Density and Light Quality for Increasing Radiation-Use Efficiency in Dwarf Tomato under LED Light at the Vegetative Growth Stage
Source: Plants (Basel). 2021 Dec 31;11(1):121. doi: 10.3390/plants11010121 (PMC8796024; doi:10.3390/plants11010121)
Supplement: Supplementary file 1 [file plants-11-00121-s001.zip › plants-1505702-supplementary.pdf]

Supplementary Materials

# Optimization of Photosynthetic Photon Flux Density and Light Quality for Increasing Radiation Use Efficiency in Dwarf Tomato under LED Light at the Vegetative Growth Stage

**Xinglin Ke [1], Hideo Yoshida [1], Shoko Hikosaka [1] and Eiji Goto [1,2,*]**

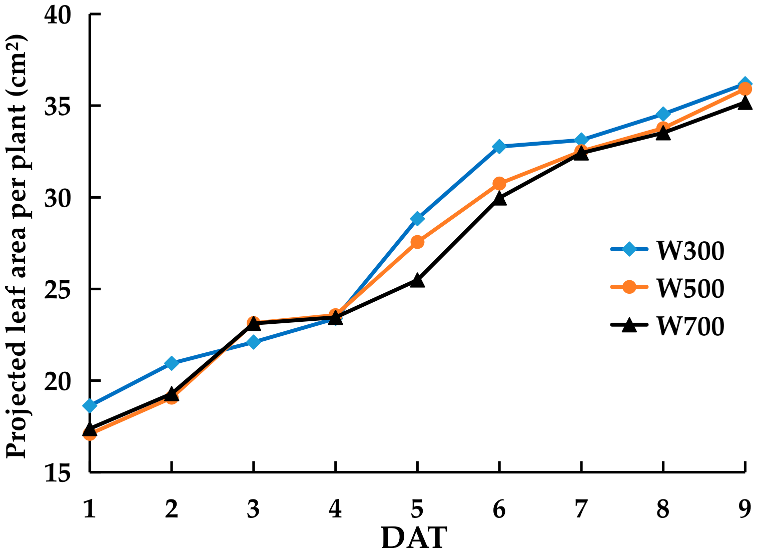


**Figure S1.** Effect of photosynthetic photon flux density (PPFD) on the projected leaf area (PLA) per plant of 'Micro-Tom' in Experiment 1. DAT represents days after treatment. Photographs of the canopy were taken from the top every day, and PLA per plant was determined from the photographs from 1 to 9 DAT. W300: 300 μmol $m^{-2}$ $s^{-1}$ PPFD, W500: 500 μmol $m^{-2}$ $s^{-1}$ PPFD, W700: 700 μmol $m^{-2}$ $s^{-1}$ PPFD.

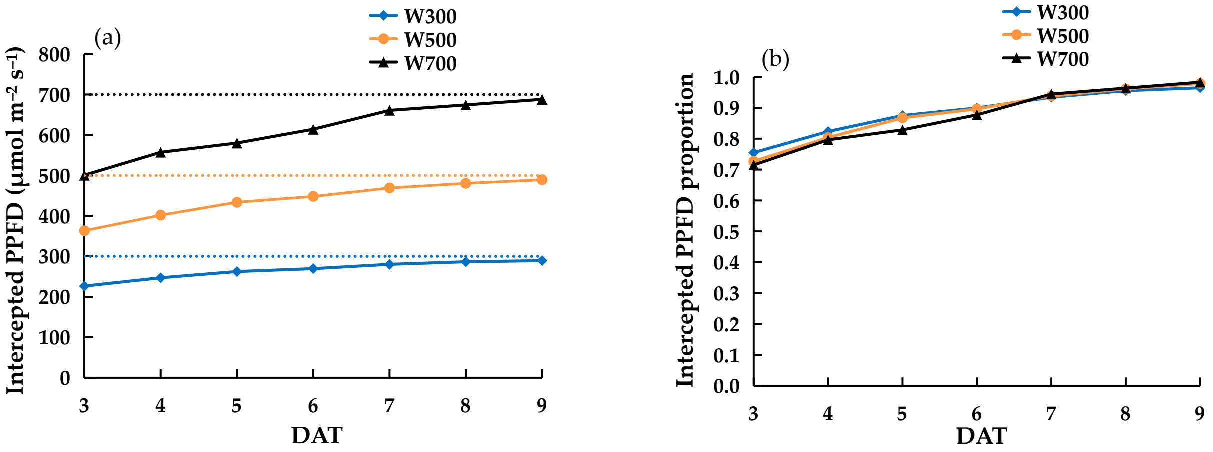


**Figure S2.** Effects of PPFD on the daily average of intercepted PPFD **(a)** and daily average of intercepted proportion **(b)** of the canopy in 'Micro-Tom' in Experiment 1. The intercepted PPFD of the canopy was calculated as the difference between the average PPFD on the top and bottom of the canopy. The intercepted PPFD proportion was calculated as the ratio of the intercepted PPFD to the average PPFD above the top of the canopy. DAT represents days after treatment. W300, W500, and W700 denote 300, 500, and 700 μmol $m^{-2}$ $s^{-1}$ PPFD, respectively.

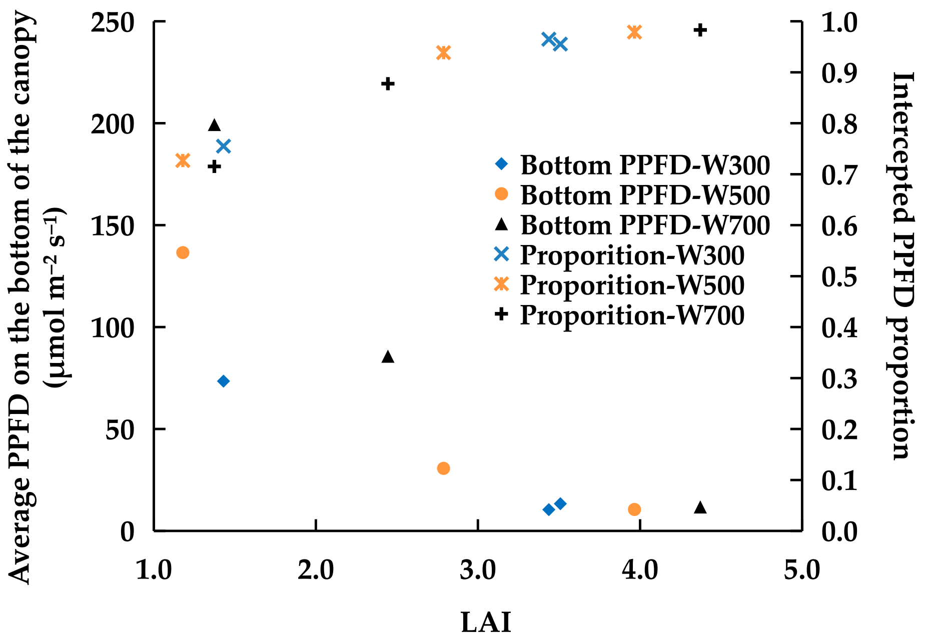


**Figure S3.** Relationships between leaf area index (LAI) and average PPFD on the bottom of the canopy and intercepted PPFD proportion under different PPFD in Experiment 1. LAI was defined as the ratio of leaf area to cultivation area. The intercepted PPFD proportion was calculated as the ratio of the intercepted PPFD to the average PPFD above the top of the canopy. W300: 300 μmol $m^{-2}$ $s^{-1}$ PPFD, W500: 500 μmol $m^{-2}$ $s^{-1}$ PPFD, W700: 700 μmol $m^{-2}$ $s^{-1}$ PPFD.

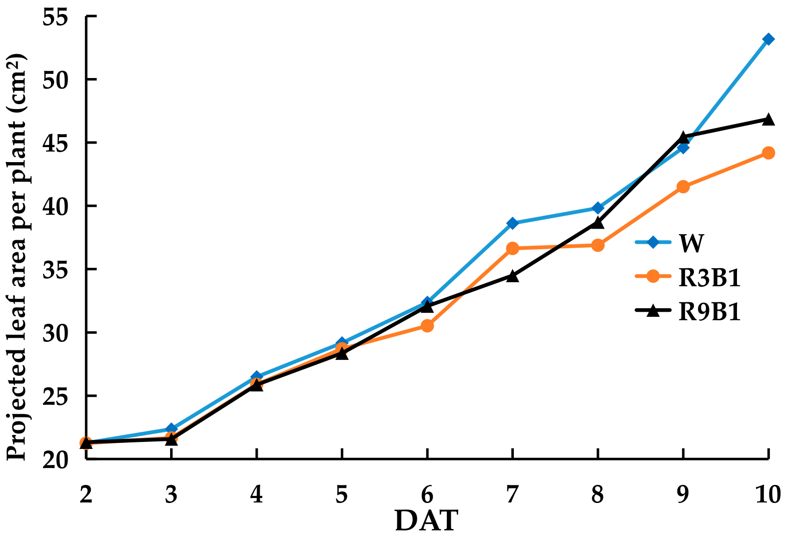


**Figure S4.** Effect of light quality on the PLA of 'Micro-Tom' in Experiment 2. DAT represents days after treatment. Photographs of the canopy were taken from the top every day and PLA per plant was determined from the photographs from 2 to 10 DAT. W: white light; R3B1: red/blue ratio = 3; R9B1: red/blue ratio = 9.

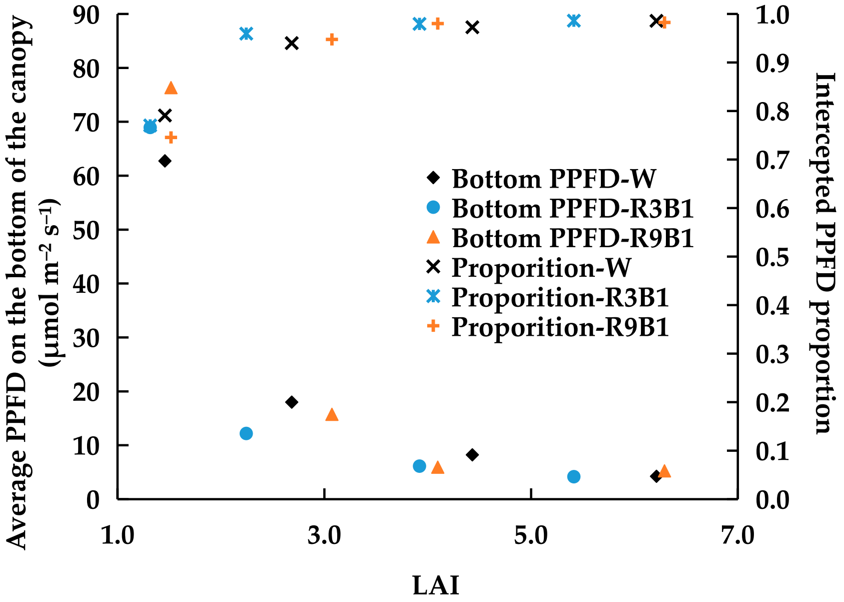


**Figure S5.** Relationships between LAI and average PPFD on the bottom of the canopy and intercepted PPFD proportion under different light qualities in Experiment 2. LAI was defined as the ratio of LA ($cm^2$) to cultivation area ($cm^2$). The intercepted PPFD proportion was calculated as the ratio of the intercepted PPFD to the average PPFD above the top of the canopy. W: white light; R3B1: red/blue ratio = 3; R9B1: red/blue ratio = 9.

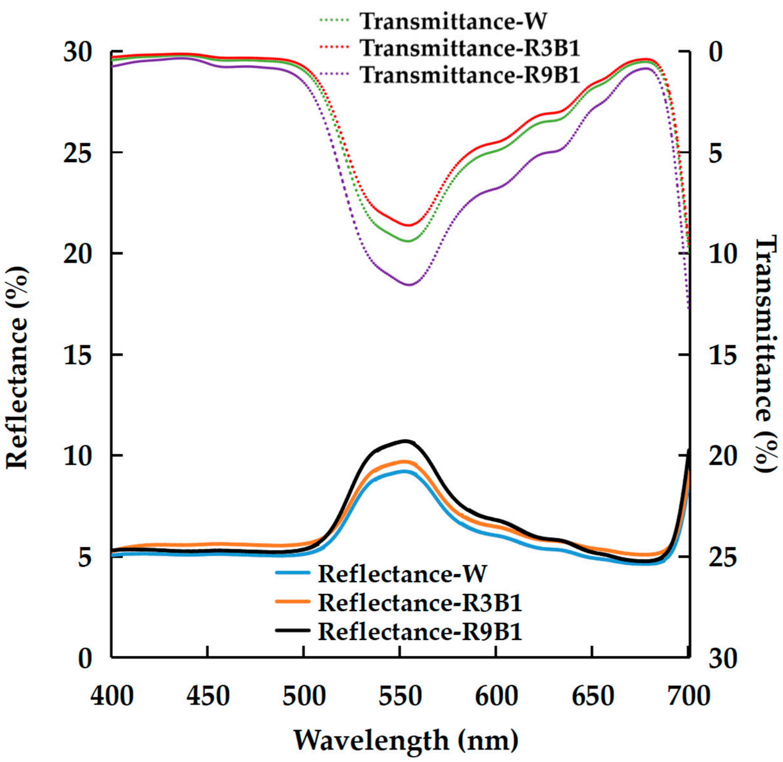


**Figure S6.** Effects of light quality on the reflectance and transmittance spectra of leaves in 'Micro-Tom' 10 DAT in Experiment 2. The range of the measured light spectrum was 400–700 nm. Each value represents the average of four plants. The PPFD of three treatments was set at 300 μmol $m^{-2}$ $s^{-1}$. W: white light; R3B1: red/blue ratio = 3; R9B1: red/blue ratio = 9.

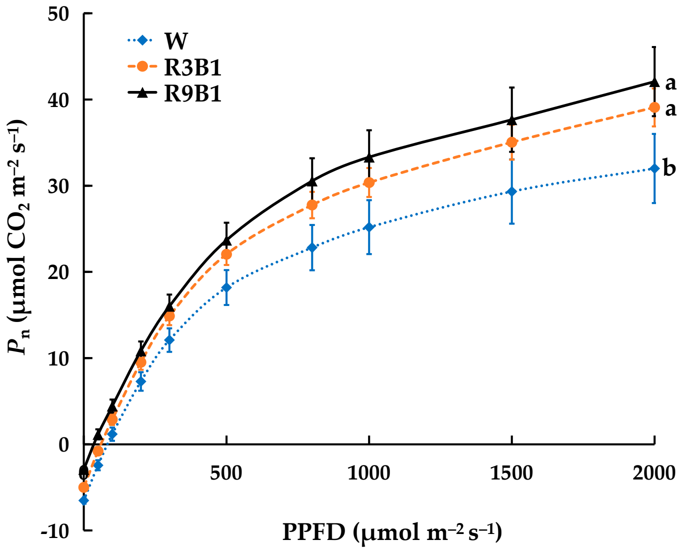


**Figure S7.** Light response curve of net leaf photosynthetic rate in 'Micro-Tom' 11 DAT in Experiment 2. Each value represents the average of three plants. Error bars represent ± standard error. Different letters indicate significant differences among the treatments based on Tukey-Kramer's test at $p < 0.05$. W: white light; R3B1: red/blue ratio = 3; R9B1: red/blue ratio = 9.

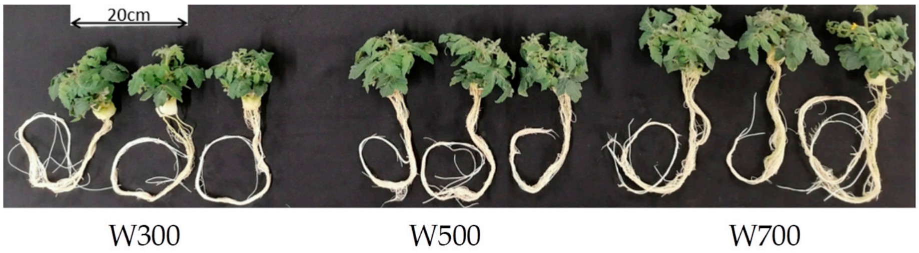


**Figure S8.** 'Micro-Tom' seedlings grown under different PPFDs 10 DAT in Experiment 1. W300: 300 μmol $m^{-2}$ $s^{-1}$ PPFD, W500: 500 μmol $m^{-2}$ $s^{-1}$ PPFD, W700: 700 μmol $m^{-2}$ $s^{-1}$ PPFD.
